# Challenging Diagnosis and Management of an Ovarian Cyst Torsion in a Postmenopausal Woman: A Case Report

**DOI:** 10.7759/cureus.47693

**Published:** 2023-10-25

**Authors:** Sara Ali, Anupama V Dhobale, Minal A Kalambe, Nandkishor J Bankar, Anand M Hatgaonkar

**Affiliations:** 1 Obstetrics and Gynaecology, Datta Meghe Medical College, Datta Meghe Institute of Higher Education and Research (Deemed to be University), Wardha, IND; 2 Microbiology, Jawarhal Nehru Medical College, Datta Meghe Institute of Higher Education and Research (Deemed to be University), Wardha, IND; 3 Radiodiagnosis, Datta Meghe Medical College, Datta Meghe Institute of Higher Education and Research (Deemed to be University), Wardha, IND

**Keywords:** benign tumors, ovarian epithelial neoplasms, cysts, ovarian torsion, ovarian masses

## Abstract

Ovarian masses are rare in the postmenopausal age group, and ovarian torsion is a gynecological emergency. We present a case report of a 63-year-old postmenopausal woman who presented a massive abdominal mass with pain that gradually increased during the previous 12 months. A contrast-enhanced computed tomography scan of the abdomen and pelvis suggested a 16.6 cm × 14 cm × 13 cm originating from the right ovary. Total abdominal hysterectomy, bilateral salphingo-oophorectomy, and partial omentectomy were performed in an emergency as the patient’s symptoms worsened. A massive cyst was visualized from the right ovary, which had undergone a torsion of three turns. Histopathological analysis revealed a serous cystadenoma. The twisted ovarian cyst typically manifests as an acute abdomen, although there are cases where this presentation can cause a significant delay in diagnosis. Therefore, high clinical suspicion is often necessary to prevent morbidity and mortality.

## Introduction

Ovarian torsion is a gynecological emergency that affects females of all age groups [1]. When the adnexal supporting organ rotates entirely or partially, the ovary experiences ischemia alterations. There are fewer isolated torsions involving either the ovary or fallopian tube, and torsions affect both structures more frequently [2]. Torsions have also been found in para-tubal or para-ovarian cysts [3]. It is vital to identify potential problems early and seek treatment promptly to protect the proper functioning of the ovaries and fallopian tubes and avoid serious health complications [4].

Cysts with a diameter of more than 10 cm on a radiographic scan or extending above the umbilicus were called huge or giant ovarian cysts. 60% of all ovarian cancers and 40% of benign tumors are ovarian epithelial neoplasms. There are two kinds of benign ovarian cysts, serous and mucinous, that are oversized and cause particular manifestations, demanding surgical intervention [5]. During the reproductive years, serous tumors are quite common, with half of all cases occurring before the age of 40. Fortunately, most of these cysts are benign, with a relatively low risk of being malignant between 7-13% for premenopausal women and 8-45% for postmenopausal women [5].

## Case presentation

A 63-year-old woman, para 2 live 2, a postmenopausal woman married for 38 years, whose bilateral tubal ligation was performed 20 years ago, visited our outpatient department with a complaint of a massive abdominal mass that had been gradually increasing over the previous 12 months. A dull and aching lower abdominal pain followed the swelling. She experienced abdominal fullness, which caused a loss of appetite and insomnia due to discomfort. The patient had a known case of hypertension. Due to a poor socioeconomic level, the patient did not seek medical assistance earlier, despite all these symptoms. Her relative took her to the hospital when she began to experience dull, painful abdominal pain and a decreased appetite. There was no family history of breast or ovarian cancer. On general examination, she was of average build and weighed 68 kg. The patient had mild pallor, was afebrile, had a blood pressure reading of 160/94 mm Hg, and respiratory and cardiovascular system examinations were within normal limits. Her per-abdominal examination revealed a distended abdomen with a girth of 108 cm and a dull note all over the abdomen on percussion. The pelvic mass extended to the umbilicus and corresponded to a 24-week gravid uterus. On vaginal examination, the uterus was normal in size, and the cervix and vagina were healthy. Radiological ultrasound of the pelvis and abdomen revealed an extensive, well-defined cystic lesion measuring around 13.8 cm × 12.4 cm × 15 cm. Doppler flowmetry was normal.

**Figure 1:**
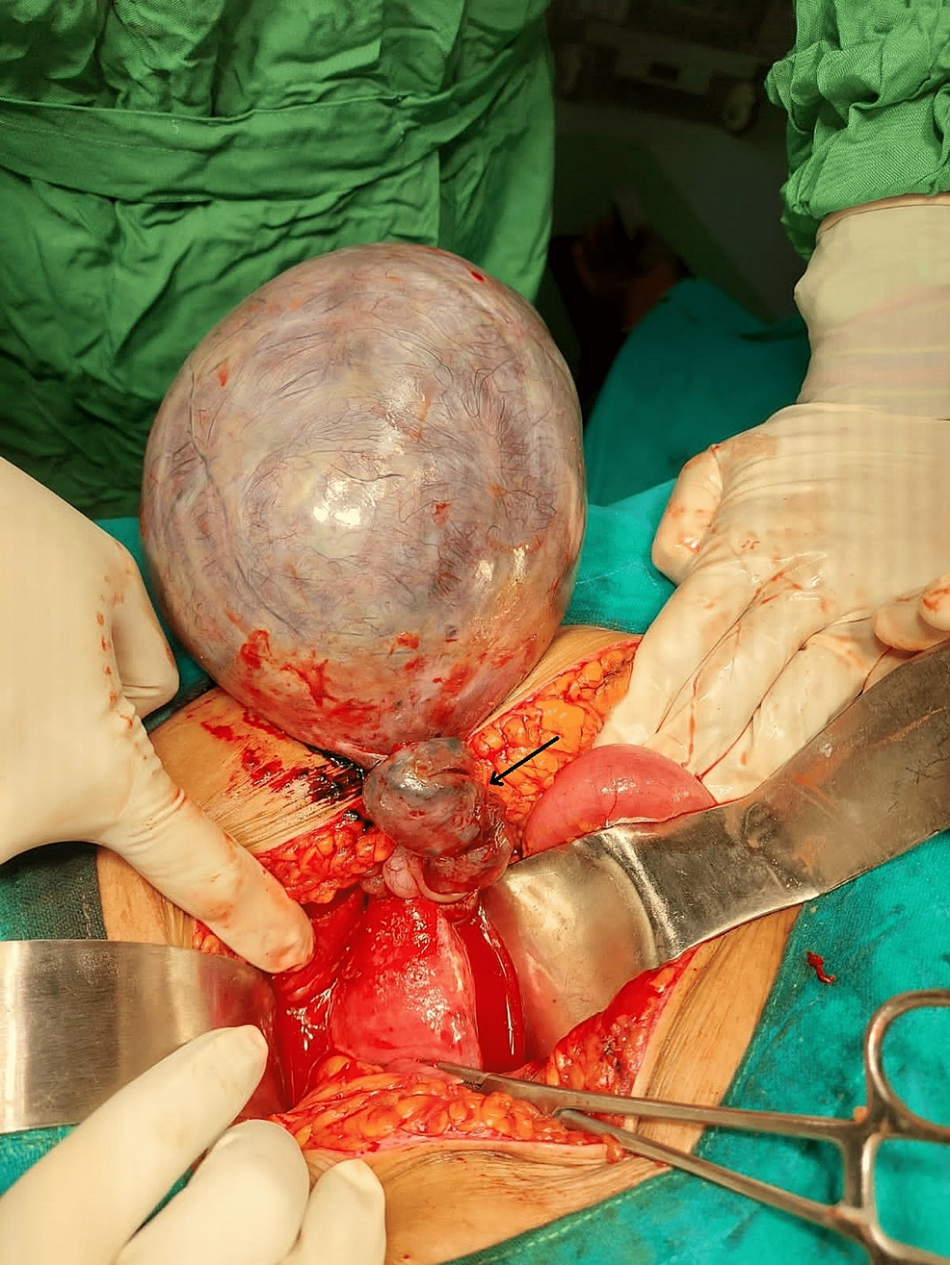
Photograph of cyst arising from right ovary with torsion of three turns The black arrow shows turns of ovarian torsion

A contrast-enhanced computed tomography scan of the abdomen and pelvis suggested a cystic lesion of 16.6 cm × 14 cm × 13 cm in the lower abdomen with a thin-walled, solid-enhancing eccentric component, as well as calcification originating from the right ovary. The left ovary and uterus appeared normal. The CA-125 is valued at 50.4 IU/ml, and the CA-19.9 is valued at 28.2 IU/ml. In an emergency, a laparotomy was performed for exploration as the patient’s symptoms worsened. A massive cyst from the right ovary was visualized and had undergone torsion of three turns (Figure 1), which measured 15 cm × 12 cm and weighed 326 g. 300 cc of blood-stained fluid from the cyst was drained and sent for cytology. The left ovary and uterus appeared normal with a smooth surface. Mild ascites were present, an omentectomy was performed, and samples were sent for histopathological examination. No group of lymph nodes was palpable. Operative findings were suggestive of a benign nature.

The procedure involved a whole abdominal hysterectomy, a bilateral salphingo-oophorectomy, and a partial omentectomy. The postoperative period was uneventful, and the patient was discharged on the ninth day of surgery. The results of the histopathological analysis pointed to serous cystadenoma, and the omental biopsy was negative for malignancy.

## Discussion

Large ovarian cysts are usually not malignant, and their histopathological analysis typically indicates them to be either serous or mucinous [5]. Postmenopausal women rarely suffer from adnexal torsion, but it has a very distinct presentation when it does occur. It is important to note that in postmenopausal women with ovarian tumors, roughly 30% of them are cancerous. Furthermore, as women age, the risk of malignancy increases. Due to these factors, it is crucial to understand that the risk of a malignant adnexal mass is higher than the possibility of torsion [6,7].

In our case, there was a 63-year-old postmenopausal para 2 live 2 woman who experienced a pelvic mass for 12 months with other associated symptoms like pain in the abdomen, fullness of the abdomen, and decreased appetite. Total abdominal hysterectomy and bilateral salphingo-oophorectomy were performed with the removal of a giant twisted right ovarian serous cystadenoma measuring 20 cm × 12 cm × 15 cm successfully.

Diagnosing ovarian torsion is challenging, but it is essential to scrutinize the symptoms, especially if the lower abdominal pain is sudden. We can identify ovarian cysts with the help of pelvic ultrasonography. Surgery is the primary approach to diagnosing and treating ovarian torsion. Preferential treatment options include detorsion, oophorectomy, or ovarian cystectomy [8]. Adnexal torsion is most common in the reproductive age group but is not very common in postmenopausal women, with an incidence of about 2.7% [9].

The ovarian vascular pediculus can rotate entirely or partially, obstructing blood flow, and is known as an ovarian cyst torsion. Any age can experience ovarian cyst torsion, although women in their 20s and 30s are more likely to experience it [10]. According to the report of Lucchetti et al. [11], in the case of adnexal torsion in premenarchal girls, the treatment aims to preserve the ovaries, and detorsion and oophoropexy can be performed. Cysts in the abdomen that are larger than 5 cm run the risk of torsion. Pregnancy, ovarian stimulation, a history of abdominal surgery, and tubal ligation are some additional risk factors for ovarian cyst torsion [12,13]. Torsion of an ovarian cyst is linked to ovarian diseases such as dermoid cysts, which increase the risk.

Metastases from the breast, gastrointestinal tract, or other rare primary organs cause up to 30% of ovarian tumors. Most of these Krukenberg tumors are in the upper and lower gastrointestinal tracts. Some centers recommend routine evaluation of these locations in women arriving with giant ovarian tumors due to the simplicity of endoscopic evaluation [14]. In postmenopausal women with a malignant tumor, laparotomy is frequently performed, considering staging surgery, while laparoscopic surgery is performed more frequently in women of reproductive age [7]. The incidence of adnexal torsion is very low in postmenopausal women, so the diagnosis is often missed. It can result in more severe outcomes due to delayed diagnosis, as the history and examination are less reliable in such patients. Ovarian serous cystadenoma is the most common benign ovarian epithelial neoplasm, and diagnosis is confirmed after a histopathological examination [15]. When a serous cystadenoma grows to more than 3 cm, patients may experience dull aching pain, intestinal and bladder problems, and pressure symptoms such as frequent urination and constipation. However, those with smaller cysts usually do not show any symptoms. To diagnose the condition, physicians can perform a bimanual examination, imaging studies like ultrasound, computed tomography, or magnetic resonance imaging of pelvic organs, and blood tests to check for cancer markers. The best way to treat serous cystadenoma is through surgery to remove the tumor, ovary, appendages, or uterus with appendages. It is essential to seek medical attention as soon as possible to prevent the disease from progressing [16-18].

## Conclusions

An emergency laparotomy was performed as symptoms worsened. During the procedure, we ruled out the possibility of malignancy and performed a total abdominal hysterectomy, bilateral salphingo-oophorectomy, and omentectomy. Twisted ovarian cysts usually manifest as an acute abdomen and are rare in postmenopausal women, although there are instances where this presentation can cause a significant delay in diagnosis. Imaging studies such as ultrasound and CT can aid in diagnosing ovarian cyst torsion. Treatment of ovarian cystadenoma depends on its size, location, and symptoms. Therefore, high clinical suspicion is often necessary to prevent morbidity and mortality.
